# The experiences of physical therapists delivering a very low energy diet and exercise intervention for weight loss in people with knee osteoarthritis: A qualitative study

**DOI:** 10.1016/j.bjpt.2025.101172

**Published:** 2025-02-08

**Authors:** Kim Allison, Rachel K Nelligan, Belinda Lawford, Sarah E Jones, Rana S Hinman, Jesse Pardo, Jonathan G Quicke, Priya Sumithran, Jodie Prendergast, Elena S George, Melanie A Holden, Nadine E Foster, Kim L Bennell

**Affiliations:** a Centre for Health, Exercise and Sports Medicine, Department of Physiotherapy, The University of Melbourne, Melbourne, Australia; b STARS Education and Research Alliance, Surgical Treatment and Rehabilitation Service (STARS), The University of Queensland and Metro North Health, Brisbane, Australia; c School of Medicine, Keele University, Staffordshire, United Kingdom; d Department of Surgery, Central Clinical School, Monash University, Melbourne, Australia; e Department of Endocrinology and Diabetes, Alfred Health, Melbourne, Australia; f Medibank Private, Melbourne, Australia; g Institute for Physical Activity and Nutrition, School of Exercise and Nutrition Sciences, Deakin University, Melbourne, Australia

**Keywords:** extended scope practice, OA, osteoarthritis, physical therapy, weight management

## Abstract

**Background:**

Exercise and weight loss are core treatments for knee osteoarthritis (OA). While physical therapists are considered well placed to engage in weight management alongside exercise (not replacing dietician care), evidence surrounding physical therapist-delivered weight loss interventions is sparse.

**Objective:**

To understand the experiences and perceptions of physical therapists delivering a very low energy diet (VLED) and exercise intervention to people with knee OA and overweight or obesity in a randomized controlled trial (RCT).

**Methods:**

Qualitative interview study involving six physical therapists (mean age 34 years, median experience 3.5 years) who completed 20 hours of training and delivered a six-month diet+exercise intervention via telehealth to people with knee OA and overweight or obesity in the POWER RCT. Physical therapists participated in semi-structured individual telephone interviews on completion of RCT involvement. Data were audio-recorded, transcribed, and independently thematically analysed by two researchers.

**Results:**

Three themes emerged. Physical therapists felt that delivering a weight loss intervention alongside exercise *enabled holistic OA management.* Enacting this role within a supportive research environment was considered unique and physical therapists felt the *training and resources facilitated care*. They felt that, with training, *weight loss may be within scope of physical therapy practice* for some patients with less complex needs although required alternate models of care including extended consult times, medical or dietician oversight and acknowledging physical therapist supervised weight loss would not be appropriate for all patients.

**Conclusion:**

Findings provide preliminary insight into potential barriers and facilitators to, and acceptability of, physical therapists delivering a VLED and exercise intervention for weight loss in people with knee OA and overweight or obesity.

## Highlights

• Physical therapists felt adding a diet to standard exercise intervention enabled care.
• Physical therapists were grateful for trial support when overseeing a diet intervention.
• They felt weight loss may be within scope of practice if other experts were involved.

## Introduction

The chronicity and complexity of obesity as a disease is well established,^1^ as is the relationship between obesity and non-communicable diseases including osteoarthritis (OA).^1^ With more than 60 % of adults living with overweight or obesity,^2^ governments and policy makers are facing the challenge of obesity-related diseases negatively impacting healthcare services and workforce capacity.^3^ The World Health Organization's recently released health service delivery framework for the prevention and management of obesity advocates for reorganization of obesity support amongst the clinical community with a focus on primary healthcare contacts as an entry point for the integration of obesity support services.^4^ While successful long-term weight loss is extraordinarily challenging due to physiological, neurohormonal, cultural, and behavioural factors that are still not yet fully understood,^5^ lifestyle interventions including diet and exercise are currently recommended as first line treatment.^4^^,^^6^ The dietetics profession is at the forefront of lifestyle weight loss interventions, but from a workforce capacity perspective are outnumbered by other health professions with synergistic expertise in exercise such as physical therapists by up to 4:1.^7^ Innovative models of care delivery that expand practice roles of healthcare practitioners may help increase access to effective lifestyle intervention support for people with overweight or obesity.

Physical therapy is one allied health profession which is well placed to synergistically engage in health behaviour support and exercise for weight loss for some patients with overweight or obesity,^8^ not replacing specialized dietician care. Physical therapists develop strong therapeutic rapport,^9^ a critical element of successful health behaviour change support interventions^10^ and clinical obesity management guidelines.^4^^,^^6^ Further, they are considered credible experts in health promotion and exercise prescription,^11^ which, along with dietary treatments, are the fundamental elements of lifestyle interventions for obesity.^4^^,^^6^ However, despite professional advocacy,^12^ weight loss has not historically been part of physical therapy practice, and as such, upskilling is required.^13^

Our team recently conducted (to our knowledge) the first randomized controlled trial (RCT) to evaluate the safety and efficacy of a physical therapist-delivered weight loss intervention in any patient population, using knee OA and overweight or obesity as an exemplar (POWER RCT).^14^^,^^15^ Delivered via telehealth, the intervention included a very-low energy diet (VLED) alongside exercise. Results showed it was effective for weight loss and improvement in pain and function, with participants in the intervention group losing an average of 8 % body weight over six months, meeting the target of 5 % body weight loss. As this is a new treatment model, further research to understand its acceptability and feasibility is warranted.

The research question was:

What are the experiences and perspectives of all six physical therapists involved in the POWER RCT delivering a VLED and exercise intervention to people with knee OA and overweight and obesity?

## Methods

### Design

Qualitative interview study based on an interpretivist paradigm where knowledge about a phenomenon is developed through gathering perceptions and interpretations of those who experience it.^16^ This study was nested within the POWER RCT (registered with Clinicaltrials.gov, NCT04733053, approved by the University of Melbourne Human Research Ethics Committee, HREC 1,955,042) that investigated the effects of a 6-month physical therapist-delivered VLED plus exercise program compared to a physical therapist-delivered exercise program alone on weight loss and knee OA symptoms.^14^ Reporting complies with the Consolidated Criteria for Reporting Qualitative Research checklist.^17^ All participants provided informed consent prior to interview.

### Participants

All physical therapists (n=6) in the RCT were invited to participate in interviews. This sample was deemed adequate based on the concept of information power^18^ which suggests that the more information the sample holds, relevant for the actual study, the lower the number of participants needed. The RCT protocol has been published.^14^ Briefly, musculoskeletal physical therapists currently registered to practice with the Australian Health Practitioner Regulation Agency working in private practice in Victoria (Australia) were recruited via our clinician network and Australian Physiotherapy Association advertisements. All recruited physical therapists delivered interventions in both trial arms (VLED plus exercise and exercise only).

### Interventions

#### Diet + exercise intervention

In brief, the Diet + exercise program involved the physical therapist delivering six individual videoconferencing consultations over six months. Initial consultation length was 75 minutes (30 minutes for the exercise component, 45 minutes diet component), subsequent consultations were 50 minutes (20 minutes exercise component, 30 minutes diet component). Physical therapists were provided hard copies of a “Physiotherapy Manual” detailing intervention/study protocols and all RCT participant resources (e.g., “Weight management activities booklet”), to facilitate consultations. Physical therapists used bespoke semi-structured electronic consultation notes containing consultation prompts and checklists to enhance protocol fidelity.

For the exercise component, physical therapists prescribed a home strengthening exercise program, including 5–6 lower limb exercises, selected from an established program,^14^ prescribed at a moderate intensity (self-perceived effort of ≥5 out of 10 (hard) on a modified Borg Rating of Perceived Exertion scale^19^) performed 3 days/week. RCT participants were provided a hard copy “Exercise Booklet”. Physical therapists also supported participants to develop a personalized, progressive physical activity plan.

The diet intervention included three phases. Phase 1 (0–12 weeks, Sessions 1–3 ± 4) ‘Weight loss’ phase: aim ≥10 % body weight loss following a VLED including two meal replacement products per day. Participants prepared their third meal and asked to ensure it was low carbohydrate, low fat, and included high-quality protein (e.g. meat/fish/vegetables/eggs/tofu) maintaining a daily total caloric intake of around 800 kcal (carbohydrate intake ≤50–60g per day). Participants were provided Optifast (Nestlé Health Science, Rhodes, Australia) or Optislim (Optipharm, Australia) meal replacements for 14 weeks, at no cost. Phase 2 (‘Transition’, Sessions 4/5, once 10 % weight loss was achieved or at 12 weeks): physical therapists supported participants in the decision to transition off the VLED (one meal replacement, reintroducing low glycemic index carbohydrates). The aim of Phase 3 (‘Weight maintenance’, end of transition onwards) was to adopt a healthy eating plan in concordance with the principles of the Commonwealth Scientific and Industrial Research Organisation (CSIRO) Total Wellbeing diet.^20^ During consultations, physical therapists used motivational interviewing techniques to support weight loss self-efficacy and motivation, and to help participants overcome any barriers to dietary and exercise adherence.^15^ Physical therapists referred to sections in the provided participant booklets including guidance to set realistic goals; keeping a food diary; identifying a support person; developing strategies to deal with challenging situations; monitoring and mindfulness of hunger levels. Education topics such as healthy foods and portion sizes were also discussed. Physical therapists were able to contact the research team (including the dietician researcher) for any concerns regarding diet prescription or monitoring throughout the trial.

#### Physical therapy mandatory training

Physical therapists were paid to undertake mandatory training (total time commitment of ∼20 hours) prior to being allocated a trial participant. Training included completion of a bespoke self-directed e-learning course (previously described and evaluated^21^) covering best practice OA management; strengthening exercise/physical activity prescription; link between overweight/obesity and OA; weight loss conversations; weight stigma in physical therapy; background to the VLED and structured trial diet intervention protocol including safety issues and behaviour change support techniques. This course has since been modified and made publicly available (www.futurelean.com/courses/eduweight). Additionally, each physical therapist conducted six training videoconference consultations of the diet program: three to a ‘mock’ patient (a researcher) and three to a ‘practice’ patient with knee OA (recruited from our consumer network). Training consultations were audio-visually recorded, and competence/fidelity assessed using a checklist of 60 items and feedback obtained from the practice patient. Subsequently, the research team provided feedback to each physical therapist regarding their competence to deliver and adhere to the intervention protocol.

### Outcome measures and data collection

#### Interviews

A semi-structured interview guide was developed (Table 1) (not pilot tested) based on the Theoretical Framework of Acceptability (TFA).^22^ All individual telephone interviews were conducted by the same researcher (RKN, a physical therapist and researcher identifying as female trained in qualitative methodologies), who was not involved in the RCT and did not know the physical therapist participants. Physical therapist participants were made aware RKN was a non-practicing physical therapy researcher. Interviews were conducted when the physical therapist had completed involvement in the RCT and lasted on average 39 mins and were audio-recorded and transcribed verbatim by an external provider. Audio-recordings and transcripts were deidentified and codes used to maintain confidentiality, and data stored on a password-protected secure university server.

**Table 1:** Interview guide.

**Domain of the Theoretical Framework of Acceptability**^22^	
**Affective attitude**	Can you tell me a bit about why you were interested in applying for the study? As a physical therapist** how did you feel about the prospect of delivering a weight loss program? Before being involved in the study, what kind of experience, if any, did you have with providing weight loss advice for your own patients? *Had you heard of a very low calorie diet before? What were your perceptions about it?*
**Burden**	What were the advantages, as a physical therapist, about delivering a weight loss program to patients? *What were the advantages for you? What were the advantages for the patient?* What were the challenges, as a physical therapist*,* about delivering a weight loss program to patients? *What was challenging for you? What was challenging for the patient? Were there any challenges with using the very low calorie diet specifically? How could they have been avoided?* What would have made these challenges easier? *What could have been improved? What should have been included?*
**Self-efficacy**	Tell me a bit about how well you thought the training prepared you to deliver the weight loss components of the intervention effectively? *What did you like and dislike about the training program? Was there anything else you felt needed to be covered by the training?* Tell me a bit about how confident you felt in your knowledge and skills straight after the training, but before your first study patient? *What did you feel most/least confident with?* *Was there anything that could have made you feel more confident?* And tell me a bit about how confident you feel now about delivering the weight loss program, having had some experience? *Were there any other training/resources that would have been helpful for you?* Given that you are a physical therapist delivering a weight loss program, how do you think patients felt about your level of expertise? *Did you feel that this had any impact on your rapport with them?*
**Perceived effectiveness**	What were the main outcomes for your patients? *How confident did you feel that your participants would be able to maintain their weight loss? Why/why not? How could this be improved?* What were the main outcomes for you - what were the best things you felt you got out of the trial?
**Ethicality, intervention coherence, and opportunity costs**	Based on your experience in this study, what do you think about physical therapists delivering this kind of weight loss program for patients in the future – not just those with OA, but also those with other chronic conditions that would benefit from weight loss? *What would need to be changed about the program used in the study, if anything?* *Would you recommend other physical therapists use the very low calorie diet with their patients? Why/why not?* *What training/support do you think physical therapists would need?* How feasible do you think it would be to integrate this weight loss program into a physical therapist business model? *What would the challenges be – cost of consultations, duration of consultations, etc* Has your experience in this study changed, or will it change, the way you treat patients with knee osteoarthritis who are overweight? *What elements of the program do you think you will use with other patients?* *What elements of the program do you think you will not use with other patients?*
**Wrap up**	Some argue that it's beyond physical therapy scope of practice to be providing weight loss support to patients. Based on your experience in this trial, what do you think about this? *Why/why not?*

* In Australia where this study was conducted, physical therapists are referred to as physiotherapists. Participants in this study used the language physiotherapist. For clarity for the international audience, the term physiotherapist has been replaced by physical therapist.

#### Descriptive data

Quantitative data were collected via individual emails sent to each physical therapist, including age, sex, years of clinical experience at RCT commencement, and level of physical therapy qualification. Number of participants each physical therapist prescribed the VLED and exercise intervention to was extracted from RCT randomization records.

### Data analysis

An inductive reflexive thematic analytical approach was used,^23^ occurring concurrently with data collection. The TFA used to inform the interview guide was not used to guide the analysis. Transcripts were first read by RKN for accuracy and to note initial ideas. RKN and a second researcher (BJL) with qualitative experience not involved in the RCT and, not a clinician, then independently applied open coding, followed by axial coding, organizing codes into categories, prior to discussing identified topics and patterns. Further supporting credibility, transcripts were read by a third researcher KA (physical therapist who codesigned the RCT, experienced in qualitative research) who reviewed the topics identified by RKN and BJL and, in collaboration, final axial coding was conducted to identify themes and subthemes. Agreement was strong amongst researchers (RKN, BJL, KA) therefore additional input into theme development was not sought. Themes and subthemes were presented with exemplary quotes. For open coding and to enable cross-referencing with quotes, NVivo 12 software (QSR International Pty Ltd) was used. All researchers strove to be cognisant of their own personal and professional viewpoints, particularly given KA and RKN's past clinical physical therapy experience could be considered as having ‘insider status’ in the physical therapy community. KA was mindful of her assumptions that supporting weight loss in a physical therapy setting could be challenging for participants. BJL approached the data analysis with no professional scope of practice pre-conceptions, but an understanding of the intervention from previous research. RKN was mindful of her assumptions of the complexities of engaging in scope of practice roles.

## Results

All physical therapists (n=6) consented and were interviewed. Two were female (33 %), all had at least a Master's level physical therapy qualification and a median 3.5 years clinical experience (range 2.8 to 28.3 years). On average, each physical therapist prescribed the VLED and exercise program to 7.5 participants (range 6 to 9). Table 2 outlines the three themes and subthemes identified and includes supporting quotes. Themes described with subthemes in italics below.

**Table 2:** Themes, subthemes, and exemplary quotes.

Themes and subthemes	Exemplary quotes
**Theme 1: Enabling holistic OA management**	
**A one stop shop**	P5: it's effectively like a one-stop shop. And that's generalising because it's not always like that. But from an exercise, education, and weight loss management side, if a physical therapist* can provide that, they're addressing all of those things as opposed to just two of the three and they have to go elsewhere for the one of the three. P6: … the convenience of it. So, the delivery of both having their – a lot of the physical side did tie in with the dietary side as well, so being able to deliver it together and kind of combine it and justify it both with each other, then that – I felt like that was good, that was pretty efficient. P1: I think it shows a holistic management and approach…That they don't have to seek out a secondary health professional. Like if it's all in-house, then it can be – it's just easier for them. There's less conflicting views and opinions, and – well less potential for those things to occur.
**An addition to the physical therapist's toolkit**	P4: I just felt like I was being able to deliver more and be more effective in getting a good outcome for my patients. It was, like I said, an extra tool under my belt. P5: So, we have an additional intervention that we can offer them rather than sending a referral off. So, I think it's a great opportunity. P1: I think it was just another – yes, another tool to add to the toolbox in terms of sometimes strengthening's not enough.
**Physical therapists well placed to deliver weight loss**	P3: I think it really goes hand in hand with a lot of those messages that we're already delivering… I'm a big advocate for it and I think we're really well placed in the community, and with our already pre-existing knowledge base - to be able to deliver that as an intervention. P5: An advantage is that we [physio] build rapport with patients, I believe, pretty well as physical therapists. They talk to us about their pain or their problems, what they can't do. And with that rapport and trust, it can be - I sense in the patients that I work with, there was that - they felt comfortable to talk through their challenges or talk through - yeah, they needed some assistance with that side of - the diet side and the weight side of things. So I think that's another advantage. P4: Like I said, it's an important discussion to have with patients and it's obviously sometimes a bit of a difficult conversation to have…And being able to deliver that I think is a really important thing for physios to have as well, because often we are the first people, people will see. Either they'll see a GP, or they'll see a physio. And I think it's important to be able to deliver some of this information.
**Theme 2: Training and resources facilitated care**	
**Practical training appreciated**	P3: I thought the training, as I said, was broad, quite in depth in terms of what we - the knowledge we needed to take forward to be able to deliver it. So I thought it was of good quality and I certainly got a lot out of it and did feel confident having had that training to then deliver that intervention. P6: I think it was – yeah, it was covered. Yeah, and there was plenty of – I think the fact that they had the practice clients and the practice sessions and then the feedback sessions were really good, prior to it. I think that was a really good addition where they're – just because it wasn't just the information. They actually had the chance to apply it first and go from there. P2: it was all really good – I used to jokingly say to people on this trial I was a quasi-dietitian and a quasi-psychologist and a real physio, because that's sort of how I felt. I felt like I had enough information to have those other two hats on, but obviously not as confident in my delivery as I would have been delivering pure physio principles. P5: The training was thorough and of really good depth in terms of the science behind the diet as well as really thorough education on the psychological factors around weight for people. So I think the training really helped me with being a physical therapist in the study…I feel maybe a little bit more practice with them [trial patients during training] may have helped. P4: I felt like I'd learnt a lot. I wasn't 100 % confident. I think it took probably a few patients, or we did a couple of the mock trials with one of the research coordinators. I think just doing one or two sessions with them really boosted - but probably a couple of patients in, that's when I really found my groove.
**Structure protocol and support resources valued**	P2: I mean look they made it really – they made it pretty easy to deliver the program - in terms of the actual content and how it was delivered, it was very prescriptive. It was very prescriptive. It was a, “Then do this. Then do this.” Yes, you couldn't really go too far wrong with delivering the content…. so I felt like it didn't go into enough depth for me to get out of my depth. If that makes any sense? P3: The materials that we had on hand in every session that we could refer back to made it easier in terms of having that knowledge and having the confidence to deliver that, but also feeling like the patients had - or the participants had the confidence in myself as well to deliver that. We were able to build that rapport and they did feel like we had a good knowledge of those things. P1: the study's got a regimented protocol and you're just going to follow that process and trust in that process, and how it had been developed….but you kind of just didn't have that clinical experience to lean on…
**Anything deviating from protocol was difficult**	P1: it was fine if people were on – were adherent and stuck to the program and were losing weight in the sort of – at the expected rate. But it was when things got a bit off – outside of normal I'll say – or in inverted commas “normal” in terms of just if there was other underlying health conditions or if blood pressure and little – if they had, I don't know, irregular dizzy spells or some other health related conditions that needed to be considered, or if they didn't like – even just didn't like the taste and weren't as adherent to the weight loss sort of intervention. Just when it got a bit off of normal, then it just became a bit of a challenge. And that's where I just felt like it was starting to stretch the scope and the knowledge a little bit. P2: So there was the weight loss phase and the maintenance phase. Yes, so just one of the challenges was just working out when to start that, because people's own goalposts sort of changed a bit. They came in saying, “I want to lose this much weight,” and so we'd agreed on that. And then when they got to that point they would then say, “No, but I actually want to try and lose a bit more weight,” so then they would stay on the first part of the program for a bit longer before they transitioned into the second part of the program, where they were just trying to manage their weight. P5: I think time is a factor. I think you have to consider your consultation times. The nature of the sessions we would do were very time based. It was encouraged to stay within a certain time which helped me do that. But sometimes, you might need to be that slight little bit abrupt in the sense of moving on to the next question…you're trying to just move them through a particular protocol, from one thing to the next because you need to have this part ticked off, I think those elements might be a little bit different or not as well accepted by the person. And it just might not feel natural to the physical therapist.
**Theme 3: Weight loss may be within the physical therapy scope of practice**	
**With training, protocolized dietary weight loss could be within their scope**	P3: I would say with the appropriate training, it can be well within our scope and aligns nicely with what we're already providing and will only make it better at what we can deliver - the interventions that we can deliver to patients and having that in our repertoire as a strategy to use when managing these patients. P2: I think with the appropriate training, most physios would be able to do that; if they were interested. Yes, if they were interested to upskill and do the training, I think most physios could effectively deliver that sort of program. P6: I don't think it's beyond the scope as long as there's enough training. So, as long as the practitioner is confident and they're able to provide the right advice and have the right knowledge, then I don't think that's beyond the scope. P4: Not really, as long as all the training is similar to what we went through. I think the only challenges would be if there just wasn't sufficient training for it.
**Outside the research environment supports are needed**	P1: Not without some form of dietary or medical oversight. P2: I don't think it's beyond a physio's scope of practice at all. I think, sure, if they were primary contact people and responsible for the whole thing from go to whoa, maybe not. But if they're working with a doctor – if they're working with someone who does the initial screening and says, “Yes, they're OK with the current medications they're on, to be on Optifast, or something similar,” then it is not beyond the scope of physios to do the rest of it; I don't think. P6: Potentially a forum, probably. So, maybe like a dietician-run forum where they can actually contact someone if they have any questions or things that they're concerned about. Yeah, just to cross-check and make sure they're doing all the right things.
**Physical therapy-delivered weight loss isn't suitable for all patients**	P1: It's beyond our scope to individualise it. Like we just don't have the expertise and the knowledge to effectively tailor a weight loss intervention. P3: I think from a safety perspective, I think it has been proven to be safe in the right patient groups as long as they're obviously - they are the appropriate patient groups that they're choosing to use the diet on.
**Integration would be feasible**	P1: I would think you'd want a longer initial appointment. Like the structure of how the study was set up, in terms of the long initial to kind of just set the scene and go through everything, I think you would need capacity to do that. And so in a clinic that's – for example, our clinic was running half-an-hour for all appointments; for initials and reviews…you can easily book a long consult and take up double the time…I think the cost would stay the same, like the physio service is what the physio service is, and what you cover in that appointment varies all the time…so this discussion around diet would just be another, as I said, tool in the toolbox. P2: Well because at the end of the day, it was just six treatments, wasn't it…I mean it slotted in – because I just work in private practice, and it was fine. My practice manager just organised the times just like they would for any other condition. So I think, yes, if adequate training was undertaken, it could easily be adopted into a physio practice… P3: It probably depends a lot on the setting of your physical therapy practice, possibly. We obviously spend already a lot of time providing education and chatting to our patients. So from that perspective, I think we're well placed, but also obviously, that's going to impact the time we spend on other interventions from a physio perspective… patient's expectation could be a challenge. There is a perception as to the role of physios in the community…and I think that could be a potential challenge or barrier to overcome. But obviously, that comes down to our ability to build good rapport and provide education to patients and be able to get them on board with what we're delivering. And that's often a common challenge for any physical therapist out there that may be even more so when you do start going down a more diet driven intervention that's delivered by a physio. I think that has the potential to be a bit of a barrier. As I said, time resourcing, absolutely. P4: I think it's pretty feasible. We've even discussed it ourselves when we were doing the study, or when I was coming on board with the study, just with our director saying - because we had this discussion saying, “Look, I think what they're doing is quite good.” And it was like, “Look, we can even say you're trained up a little bit more in this area and we can maybe actually implement some of this stuff

* In Australia where this study was conducted, physical therapists are referred to as physiotherapists. Participants in this study used the language physiotherapist. For clarity for the international audience, the term physiotherapist has been replaced by physical therapist.

Theme 1 Enabling holistic OA management

Physical therapists described the intervention as a *one-stop shop* approach to exercise, education, and dietary weight loss for people with knee OA. They emphasized the convenience of addressing all these aspects together, eliminating the need for patients to seek multiple health professionals and reducing potential conflicting treatment advice. Physical therapists described the weight loss program as an *extra tool in a physical therapist's toolkit*, enabling them to offer a new service, build rapport, and be more effective in delivering holistic patient care. They viewed physical therapists as being *well placed to deliver weight loss* as they are often among the first healthcare professionals that patients consult, and delivering dietary advice was seen as complementary to their existing health messages.

Theme 2 Training and resources facilitated care

Prior to involvement in the trial, the physical therapists had minimal experience discussing weight with their patients. Hence, the *practical training was appreciated,* especially the inclusion of practice sessions and feedback which allowed them to apply what they had learned. However, despite considering the training comprehensive, they still felt less confident in delivering the dietary component of the intervention compared to the exercise component. It was suggested that additional sessions with ‘practice’ patients during training could have been beneficial (for example 1–2 more practice patients). The *structured protocol and support resources were valued* and viewed as well-developed and trustworthy, providing clear, step-by-step guidance. This was seen as facilitatory given the physical therapists' limited clinical experience in supporting weight loss. However, *anything deviating from the protocol was difficult.* For instance, they felt they lacked the knowledge to adapt the intervention when patients experienced slower-than-expected weight loss, or unfamiliar symptoms like constipation, or low adherence.

Theme 3 Weight loss may be within the physical therapists’ scope of practice

Overall, physical therapists believed that, *with* training (like that provided but with additional ‘practice’ patients)*, protocolised dietary weight loss could be within their scope* of practice, seeing it as a natural extension of their existing role and aligning with care they already provide. Indeed, most had incorporated learnings from the RCT into their clinical practice. For instance, discussing weight with patients, recommending online dietary resources, and applying motivational interviewing techniques during patient encounters. But they recognized that *outside the research environment, supports (or safeguards) would be needed* for physical therapists to deliver a dietary intervention safely and effectively. Potential support options described included initial assessments by medical or dietetic professionals to ensure suitability and ongoing oversight and/or a forum, potentially run by a dietitian, where physical therapists could cross check process and seek clinical guidance. They also emphasized that a *physical therapist-delivered dietary intervention for weight loss isn't suitable for all patients,* for example, those with complex needs would be outside the physical therapists’ scope of practice and should be referred on for tailored dietary and weight loss advice (e.g., those with comorbid conditions such as uncontrolled diabetes). Finally, physical therapists believed that integrating such a program into the private practice business model *would be feasible* (if physical therapists had interest in upskilling). They acknowledged that initial consultation length would need to be extended (e.g., offering a double consult), and some believed there would be no impact on service costs, while others suggested a fee increase would be appropriate to reflect additional expertise.

## Discussion

This qualitative interview study explored the experiences and perspectives of six physical therapists who delivered a novel VLED and exercise intervention, for people with knee OA and overweight or obesity. Physical therapists felt that the dietary component was a key addition to their treatment toolkit, enabling them to provide holistic patient care aligned with clinical guidelines. They also felt that, as primary care providers of OA management, they were well placed to deliver the combined intervention. However, they also recognised that implementing a dietary intervention outside of a supportive RCT setting would require further practical training (additional practice patients), support from medical or dietician practitioners, and/or alternative models of care. To our knowledge, this is the first study to explore the experiences and perspectives of physical therapists who have delivered such an intervention to people with any chronic condition and overweight or obesity. Together with qualitative research from patients receiving the intervention,^24^ our findings provide preliminary insight into the barriers, facilitators, and acceptability of a physical therapist-delivered VLED in a real-world setting.

Physical therapists in this study believed the combined dietary and exercise intervention enhanced their clinical care of people with knee OA. A sentiment that is echoed in our study exploring the experiences of a subgroup of people with knee OA who received the intervention in the RCT.^24^ These RCT participants cited the primary advantage of the program being the need to only see one healthcare professional for both diet and exercise, which they saw as complementary and effective for weight loss and knee OA symptom management.^24^ However, some participants felt that their physical therapist had a superficial level of knowledge and relied too heavily on the protocolized trial resources.^24^ In contrast, in this study, physical therapists cited the protocolized intervention as an advantage of the program, while acknowledging that they did lose confidence when a patient's response or needs deviated from the protocol (e.g., unexpected symptoms like constipation or dizziness). Indeed, VLEDs utilizing meal replacements are, by nature, highly protocolized.^25^ This structure may mitigate the need for extensive nutritional and dietary expertise, for patients without specific dietetic needs (e.g. dietary restrictions/intolerances) and may be a practical option for physical therapists where in-depth nutritional and dietary expertise are beyond the current scope of physical therapy practice, entry to practice degrees, and our training model. Given the prevalence of comorbidities with both OA^26^ and obesity,^1^ there are complex subgroups of patients who will require more specific medical supervision and dietary advice than physical therapists alone can provide. We acknowledge that managing such patients is currently outside a physical therapists’ scope of practice, as did physical therapists in the present study.

Physical therapists greatly appreciated delivering the intervention in the supportive research environment as it gave them confidence to deliver the dietary component despite having limited experience. They relied heavily on the physical therapist and patient trial resources and were reassured knowing that they could contact the research team (including the dietician researcher) for guidance, if needed (although during the RCT few did this). In addition, in the RCT, people were excluded from participating if they were deemed not suitable for a VLED without medical supervision. This included people with Type I diabetes, Type II diabetes requiring medication aside from Metformin, warfarin use, stroke or cardiac event in previous six months, unstable cardiovascular condition, fluid intake restriction, and renal pathology without general practitioner clearance.^14^ To facilitate real world implementation, physical therapists felt that similar support would be needed. For example, initial suitability screening conducted by a dietician, or a general practitioner would be needed, and ongoing medical oversight and referral preferred. Understanding which patients require specific support for weight loss beyond the scope of an individual practitioner's skills, is a fundamental competency of weight management.^27^ Physical therapists are typically skilled at triaging and referring on when indicated,^28^ and as a profession do not operate in isolation. Physical therapists frequently work alongside other allied health professionals and general practitioners,^29^ or can be connected digitally to other healthcare networks,^29^ affording opportunity for more integrated care when supporting weight loss in people with complex co-morbidities, not to replace dietician care.

Evidence has shown that both patients^11^ and physical therapists^13^ believe that supplementary training or credentialing in weight management is fundamental to the credibility of, and their confidence in, a physical therapist-delivered weight loss intervention. Physical therapists in this study undertook, on average, 10–12 hours of self-directed online learning, completing a program previously shown to be effective in increasing confidence in knowledge and skills in weight management and reducing weight stigmatized attitudes.^21^ In addition, physical therapists also conducted 10 hours of practical, synchronous ‘mock’ consultations with a researcher and recorded video consultations with “practice” patients (people with knee OA not in the RCT). This enabled real time and follow up feedback on competency and intervention fidelity. However, to provide effective dietary weight loss support outside of a research setting, physical therapists may require enhanced training. This need was recognized by the physical therapists in this study, who felt that additional practice sessions, such as 1–2 more mock consultations, would have further increased their confidence and competence. Indeed, the opportunity to practice and receive feedback is fundamental to adult learning^30^ and is frequently reported as the most beneficial component of healthcare professional training.^31^ Moreover, during the RCT, physical therapists delivered the VLED and exercise intervention to approximately 7 participants only. As the physical therapists indicated that their confidence grew as they gained more experience during the trial, it's reasonable to expect that their confidence would have further improved if they had delivered the intervention to more participants.

Several methodological considerations should be noted when applying this study's findings to other contexts. All six physical therapists, who had the experience of delivering the VLED and exercise intervention in our RCT were interviewed, however transcripts or findings were not returned to interviewees for checking. Although small, this sample was deemed adequate based on its high information power due to the narrow study aim, high sample specificity, and strong quality (clear and focused) interview dialogue produced.^18^ These therapists did not treat many individual patients; hence their experiences may have been different if they had treated more patients within the program. The majority (three) physical therapists were relatively clinically inexperienced (within five years of graduation), and none had additional training in weight management. These physical therapists applied to be part of the trial, therefore may be more interested or confident in delivering a dietary weight loss intervention. They were also paid to undertake the training and deliver the intervention. It may be that more experienced or a broader group of physical therapists would have had different perspectives and experiences with the training provided and the delivery of a dietary intervention and our findings cannot be generalized to all physical therapists. Further, weight loss support exists along a continuum, ranging from light touch interventions involving direction to resources or support through to prescribing and overseeing a tailored weight loss intervention,^8^ the latter which was investigated in this study. Further research is required to understand how and if physical therapists can deliver other forms of weight loss support. Finally, to optimize credibility of this work we included a research team with different professional research backgrounds (physical therapy, OA, consumer advocacy) and used multiple team members at each stage of analysis to scrutinize the analysis and interpretation.

## Conclusion

Physical therapists who delivered an RCT intervention consisting of a VLED and exercise program for weight loss in people with knee OA, felt that they were able to provide holistic OA care and were well placed to do so. However, while they valued the training provided within the RCT, they felt that further practical training and support would be needed if physical therapists were to play a greater role in weight management outside a supported research environment.

## Declaration of competing interest

The authors have no competing interests to declare.
